# Self-reported methods of weight cutting in professional mixed-martial artists: how much are they losing and who is advising them?

**DOI:** 10.1186/s12970-019-0320-9

**Published:** 2019-11-12

**Authors:** Sungjun Park, Michelle Alencar, John Sassone, Leilani Madrigal, Alison Ede

**Affiliations:** 1 Department of Kinesiology, California State University, Long Beach, Long Beach, CA USA; 2 Fitness & Integrated Training Laboratory and Sport Studies & Sport Psychology Laboratory, Long Beach, USA; 3 Department of Family and Consumer Science, Nutrition and Dietetics, California State University, Long Beach, USA

**Keywords:** Rapid weight loss, Weight class, Weigh-in, Body mass, Hydration and dehydration, Competition, Nutrition

## Abstract

**Background:**

Similar to other combat sports, mixed martial arts (MMA) includes divisional weight classes. The purpose of our research was to further investigate the amount of weight professional MMA fighters lost prior to weighing in for competition, their methods used to cut weight, and their sources of advice on how to cut weight.

**Methods:**

This survey was administered to 92 male professional MMA athletes. The survey questions included duration of overall weight loss prior to competition, methods of weight-cutting, and their sources of advice regarding weight cutting.

**Results:**

When comparing the number of methods of weight cutting with the source of advice, those who received their advice from social media used slightly more methods of weight cutting (*M* = 4.86, *SD* = 1.27) than those who did not (*M* = 4.02, *SD* = 1.55); t(90) = − 2.53, *p* < .05. MMA athletes that used the help of a registered dietitian nutritionist also reported using the least amount of methods for weight-cutting than any other category (*M* = 3.84, *SD* = 1.67). Those that used teammates and did not use a registered dietitian nutritionist used slightly more methods (*M* = 4.46, *SD* = 1.41) than those who used a registered dietitian nutritionist.

**Conclusions:**

The findings of this study report that professional MMA athletes do undergo rapid weight loss through various methods to make weight for competition. This study adds evidence to the literature that most professional MMA athletes undergo RWL for competition without the guidance of a registered dietitian nutritionist. It is unclear what the effect of using a registered dietitian nutritionist may have on an MMA athletes’ ability to reduce weight in a safe and effective manner. Future research should seek to investigate if employing a registered dietitian nutritionist may lead to a higher rate of success for MMA athletes to make weight, and help reduce adverse risks of RWL.

## Introduction

The sport of mixed martial arts (MMA) is rapidly growing and increasing in popularity [1, 2]. As in other combat sports, competitions in MMA are in divisional weight classes [3]. Most professional MMA organizations have a mandatory waiting period between weighing in and competition. In the weeks leading up to weigh-in, MMA athletes try to gain an advantage by manipulating their fighting weights using methods of rapid weight loss (RWL), also known as weight cutting. With gaining recognition of the sport, more athletes are turning to the MMA as a career opportunity. Most MMA athletes lose differing amounts of weight rapidly by dehydrating prior to weighing in. They then use the period between weighing in and competition to try to replenish fluids and increase body weight [4–7]. A study with 17 MMA athletes from all weight classes and expertise indicated that they lost up to 10% body mass in the week prior to weighing in [7]. A study with thirty 125 lb. to 170 lb. weight class MMA athletes indicated that they lost an average of 9 ± 2% body mass in the week prior to weighing in, and added an additional 5 ± 2% body mass in the final twenty-four hours after weighing in [5]. No studies to date have investigated these practices in only professional MMA athletes.

Researchers studying RWL and dehydration indicate the potential for physiological changes that can be detrimental to MMA performance. MMA competition is taxing on the aerobic, anaerobic, and phosphagen energy systems [2, 8]. The effects of RWL due to hypohydration include reduced blood volume, plasma volume, stroke volume, sweat rate, heat dissipation, free testosterone, and blood creatine concentrations. Dehydration increases plasma osmolarities, blood viscosities, blood urea concentrations, blood cortisol concentrations, blood ammonia concentrations, and catecholamine responses [4, 7, 9–13]. These effects from dehydration manifest as increased glycogen utilization, core temperature, and heart rate [4, 7, 9–13]. Furthermore, these physiological changes may hinder motor skills, alertness, mood, cognition, and flexibility [13–17].

The evidence regarding the effects of dehydration and weight advantages on combat sports performance is inconclusive [18–21]. A study of 40 MMA athletes who added 3.40 ± 2.2 kg or (4.4%) of body mass during a 22 hr period after weighing in indicated that 39% were still dehydrated [6]. Post weighing in, some athletes may need to replace over 10% of their body weight with fluids to be rehydrated [5, 7]. Little research has been conducted to conclude if this amount of fluid can be replaced adequately in the hours between weighing in and professional MMA competition.

When investigating the most common methods of RWL in all types of MMA athletes (amateur and professional), the five most common methods consistently reported are food restriction, increased training, use of a sauna, use of a sweat suit, and water-loading [4, 5, 23–26]. Other methods that are not as common but still present include the use of salt baths, training in heated rooms, use of laxatives, intake of diuretics, spitting, and vomiting [23, 26]. A noticeable trend has been the increased use of the method of water-loading, a method where an individual will attempt to induce excessive urine production by reducing the intake of sodium and drinking an excessive amount of water leading up to weigh-in [5]. To date, it is unknown if the methods used by only professional MMA athletes differs from responses by amateur and professionals.

While the amount of weight lost and methods of weight-cutting has been previously investigated, there has been a lack of research investigating the sources of information professional MMA athletes will use towards weight-cutting practices. In a previous study investigating weight-cutting practices of combat athletes that included Judo, Jujitsu, Karate, and Tae kwon do, only 26.1% of athletes used the guidance of a nutritionist to cut weight for competition, while 68.1% were advised from their fitness instructor, and 30.0% from friends [22]. Specifically, to MMA athletes of all levels, only 20% reported using the help of a registered dietitian nutritionist for dietary advice for cutting weight [5]. With more athletes turning to MMA as a career option, it is important to note where professional MMA athletes seek guidance. A registered dietitian’s scope of practice includes the design and implementation of nutrition strategies for optimal performance in sport [23]. In addition, a registered dietitian nutritionist evaluates and guides athletes to know when and how much to consume food and fluids to maintain a healthy body weight and composition for physical performances [23]. Other sources are common for MMA athletes including teammates, coaches, magazines, social media [3, 5, 24, 25]. Thus it may be important, for a registered dietitian nutritionist to be the most qualified professional to advise MMA athletes on completing a safe weight-cut for competition.

The purpose of the present study was to investigate the amount of weight professional MMA fighters lose prior to competition, their methods used to cut weight, and their sources of advice to cut weight. Understanding these factors is significant to professionals working with professional MMA athletes including coaches, trainers, and nutrition professionals. As the sport grows, it’s important for professionals to gain a deeper understanding of current practice in professional MMA athletes. This information can be useful so they may best guide MMA athletes on proper methods for weight reduction.

## Methods

### Subjects

A total sample of 92 male professional mixed martial arts athletes (*n* = 92) volunteered to participate in the current study. The participants were recruited through convenience sampling and reported training primarily in the states of California and New Mexico, USA. Participants also ranged in weight classes from atomweight (47.6 kg) to heavyweight (120.0 kg) and were accepted as professional if they reported to have competed in a professional MMA fight, and reported to be classified as professional status within that fight during the past year. Athletes were informed clearly of the procedures of the study, and the possible benefits and risks of the study. Written informed consent was obtained prior to participation, and this study was approved by Long Beach State University’s Institutional Review Board (IRB).

### Procedures

Upon IRB approval, a general information email about the study and athletes’ options of involvement was sent to head coaches of various MMA training facilities. Head coaches were then able to help coordinate a date when the survey could be distributed to the athletes at a convenient time. A 32-item survey was distributed to participants at their primary training location and was not validated prior to distribution. Although the survey was not a validated measure of weight cutting strategies of professional mixed martial artists, none exist to date [9]. Additionally, the assessment tool was created by a collaborative effort by sport registered dietitian nutritionists, exercise physiologists, and certified strength and conditioning specialists as a practical and easily acceptable tool for professional mixed martial artists. The survey was designed to be simple and straight-forward, with the fewest-questions possible, in order to increase the likelihood that athletes would complete the survey. This survey was distributed in-person for convenience.

Information was collected pertaining to the participants’ typical weight (when the athlete is not training for a specific event/competition) and current weight (when the athlete completed the survey). Additionally, information about weight-cutting habits were also collected, including: what weight class the participant usually competed in, if they cut weight for competition, how much weight they cut, when they would cut the most weight in relation to how many weeks out of competition, the amount of days it took to make weight, methods of weight-cutting, and their sources of advice for weight-cutting (registered dietitian nutritionist, social media, doctor, teammates, professional organizations). All participants were clearly informed of each source of advice. All participants completed the entire survey, and the completed surveys were reviewed by the research team for analysis. A cross-sectional analysis was conducted to examine the frequencies of weight-cutting methods, durations of weight-cutting, and sources of advice on weight-cutting.

### Statistical analyses

Analysis of the data consisted of descriptive statistics reflected as means (M) +/− standard deviations (SD) and frequency of responses to each item. A Spearman’s Rho correlation was conducted between the length of time and amount of weight cut, and was calculated using IBM Analytics, SPSS v24, significance set at *p* < 0.05. Due to the exploratory nature of the study, the sample size was not determined prior to data collection.

## Results

Descriptive statistics were used to determine weight-cutting behaviors of MMA athletes, and will be presented based on category.

### Amount and timing of weight cutting

Participants ranged in the length of their weight cuts prior to competition (4.66, ± 2.32 weeks). As shown on Table 1., MMA athletes reported starting weight losses between 1 to 8 weeks prior to competition. The highest frequencies indicated that athletes started cutting weight around 4 (*N* = 21, 22.8%) and 6 weeks (N = 21, 22.8%) prior to competition. Within this 4–6-week period, 39 MMA athletes reported cutting 11–26+ pounds. In addition, when MMA athletes were asked to record when they lost the most amount of weight, 30% reported 1 week prior to competition (7 days), 27.8% reported 48 h prior to competition, and 22.2% reported 24 h prior to competition.

**Table 1:** Frequency of MMA athletes that lost a certain amount of weight within a certain amount of time leading to weigh-in. N = Number of MMA athletes

	Overall %	0–2 pounds	3–5 pounds	6–10 pounds	11–15 pounds	16–20 pounds	21–25 pounds	26+ pounds
1 week prior	13.0%				40.0%	10.0%		8.7%
2 weeks prior	9.8%				25.0%	5.0%	12.5%	
3 weeks prior	5.4%					5.0%	12.5%	4.3%
4 weeks prior	22.8%		100.0%	100.0%	10.0%	30.0%	20.8%	21.7%
5 weeks prior	3.3%						8.3%	4.3%
6 weeks prior	22.8%				10.0%	35.0%	29.2%	21.7%
7 weeks prior	3.3%				5.0%	5.0%	4.2%	
8 or more weeks prior	17.4%				10.0%	10.0%	12.5%	39.1%
Don’t cut weight	2.2%	100.0%						
Total	100%	2	1	2	20	20	24	23

### Methods of weight cutting

As shown on Table 2., participants ranged in the number of methods used to cut weight (4.27 ± 1.51 methods). MMA athletes recorded a range of methods used to cut weight.

**Table 2:** Frequency (percent) of methods used to cut weight by MMA athletes in relation to amount of weight lost. *N* = Number of MMA athletes

Method	Frequency (%)	Amount of weight cut						
		0–2 lbs.	3–5 lbs	6–10 lbs	11–15 lbs	16–20 lbs	21–25 lbs	26+ lbs
Overall N	92	2	1	2	20	20	24	23
# of methods used M (SD)	4.27 (1.51)	0 (0.0)	2 (2.0)	5.50 (.71)	4.45 (1.19)	4.05 (1.15)	4.46 (1.59)	4.48 (1.50)
Food restriction	82.6%		100.0%	100.0%	85.0%	85.0%	79.2%	87.2%
Sauna	69.6%				75.0%	65.0%	66.7%	78.3%
Diuretics	2.2%						4.2%	4.3%
Sweat-suit	59.8%				65.0%	65.0%	158.3%	56.5%
Increased training	69.6%		100.0%	50.0%	80.0%	70.0%	70.8%	65.2%
Water load	72.8%			100.0%	80.0%	70.0%	79.2%	69.6%
Vomiting or laxatives	1.1%				5.0%			
Salt bath	29.3%			50.0%	20.0%	30.0%	37.5%	30.4%
Salt load	4.3%				5.0%		8.3%	4.3%
Juice cleanse	3.3%				10.0%			4.3%
Sweet sweat	15.2%				10.0%	15.0%	20.8%	17.4%
Low carb	1.1%							4.3%
Colonic	3.3%					5.0%	4.2%	4.3%
Water restrictions	2.2%				5.0%		4.2%	
Jacuzzi or hot tub	6.5%			50.0%	5.0%		12.5%	4.3%
Other (low sodium diet)	2.2%							8.7%
Other (ketogenic diet)	1.1%							4.3%

The most common methods of weight-cutting included food restriction (82.6%), water-loading (72.8%), increased training or energy expenditure (69.6%), sauna use (69.6%), and sweat-suit use (59.8%). When comparing the amount of weight cut by method, food restriction was the most frequently used by those cutting weight up to 26+ pounds.

Considering amount of weight lost was not a continuous variable, we could not calculate the mean amount of weight cut for those who used a certain method vs. those who did not use a certain method. However, when we grouped MMA athletes to those who went through a short training camp (1–4 weeks), and those who went through a longer training camp (5–8 weeks); those who went through a longer training camp were significantly more likely to use increased training or energy expenditure than those who cut weight within a short training camp, *X*^*2*^ (2, *N* = 90) = 6.373, *p* = .012.

### Sources of advice

In this study, teammates were reported as the most common source (83.7%) for advice on nutrition and weight-cutting by MMA athletes. Social media, registered dietitian nutritionists, and “other” were among the additional sources MMA athletes used. As shown on Table 3., the method of weight-cutting by food restriction had the most sources of advice. In terms of “other” sources, this included: online sources, endorsed programs, and support systems (family, friends, coaches, significant others).

**Table 3:** Frequency (percent) of MMA athletes that used a source of advice for a specific method to cut weight. N = Number of MMA athletes

Method	Source of Advice								
	Registered dietitian nutritionist	Teammates	Magazines	Doctor	Social media	Prof. orgs.	Other: Friends	Other: coach	Other
Overall N	19	77	4	0	28	2	3	4	27
Food restriction	73.7%	84.4%	100.0%		85.7%	50.0%	100.0%	100.0%	81.5%
Sauna	68.4%	72.7%	100.0%		78.5%	50.0%	66.7%	25.0%	77.8%
Diuretics		1.3%							3.7%
Sweat-suit	52.6%	62.3%	100.0%		60.7%		33.3%		55.6%
Increased training	63.2%	70.1%	50.0%		71.4%	100.0%	100.0%	100.0%	63.0%
Water load	68.4%	75.3%	100.0%		85.7%	100.0%	100.0%	100.0%	63.0%
Vomiting or laxatives		1.3%							
Salt bath	21.1%	29.9%	50.0%		39.3%	50.0%		25.0%	18.5%
Salt load	10.5%	5.2%							3.7%
Juice cleanse		1.3%			7.1%				7.4%
Sweet sweat	5.3%	16.9%	50.0%		28.6%	50.0%	33.3%	25.0%	26.0%
Low carb	5.3%								3.7%
Colonic	5.3%	3.9%	50.0%		7.1%				7.4%
Water restrictions		2.6%							7.4%
Jacuzzi or hot tub		7.8%			14.3%			25.0%	7.4%
Other (low sodium diet)	5.3%	1.3%							3.7%
Other (ketogenic diet)					3.6%				3.7%

**Table 4:** Descriptive Statistics of number of methods used by athletes that use a certain source of advice with the number of methods used by athletes that do not use a certain source of advice. N = number of MMA athletes, M = Mean, SD = standard deviation

	Source of Advice								
	Registered dietitian nutritionist	Teammates	Magazine	Doctor	Social media	Professional orgs	Other: Friends	Other: coach	Other
Overall N	19	77	4	0	28	2	3	4	27
# of methods used M (SD) who use that source	3.84 (1.68)	4.36 (1.41)	6.00 (1.41)		4.86 (1.27)	4.00 (1.41)	4.33 (1.15)	4.75 (1.71)	4.37 (1.60)
# of methods used M (SD) who do not use that source	4.38 (1.46)	3.80 (1.93)	4.19 (1.48)		4.02 (1.54)	4.28 (1.52)	4.27 (1.53)	4.25 (1.51)	4.23 (1.49)

When comparing the number of methods used to cut weight with the source of advice, those who used social media as their source of advice also used slightly more methods to cut weight (*M* = 4.86, *SD* = 1.27) than those who do did not use social media (*M* = 4.02, *SD* = 1.55); t(90) = − 2.53, *p* < .05. Additionally, those who used a registered dietitian nutritionist also used the lowest number of methods than any other category (*M* = 3.84, *SD* = 1.67). Athletes that refrained from going to teammates for advice used the lowest number of methods to cut weight (*M* = 3.80, *SD* = 1.93). Athletes that only used teammates and did not use a registered dietitian nutritionist used slightly more methods (*M* = 4.46, *SD* = 1.41) than those who used a registered dietitian nutritionist. Finally, those that used both a registered dietitian nutritionist and teammates used less methods (*M* = 3.93, *SD* = 1.44), than those who used teammates Table 4.

## Discussion

The aim of the current study was to examine the amount of weight professional MMA fighters lost, the methods used to cut weight, and the sources of advice MMA athletes used to cut weight. This study reports a high percentage of professionally classified MMA athletes do engage in RWL. In this study, 98% of professional MMA athletes recruited reported using RWL to make weight for competition. This percentage is consistent with previous literature investigating RWL habits of Brazilian MMA athletes in all competitive levels, where 95% reported RWL for competition [5]. In comparison, when investigating RWL habits of other types of combat athletes including judo, jiu-jitsu, tae kwon do, and karate, only 50% of international and 66.3% of national competitive athletes reported engaging in RWL [9]. This may indicate that the MMA athlete is more likely to engage in RWL practices than other combat athlete. Furthermore, the percentage of MMA athletes engaging in RWL practices is similar between populations of professionals and all levels.

This study also presents two main findings with regards to amount of weight lost through RWL and timing (including duration) of RWL in professional MMA athletes. First, the greatest frequency of MMA athletes engaging in RWL occurs at 4 and 6 weeks prior to competition. Second, the greatest amount of weight lost by athletes engaging in RWL occurs within the last week prior to competition, with 30% reported cutting weight 1 week prior, 27.8% cutting 48 h prior, and 22.2% cutting 24 h prior to competition. This contrast is most likely due to the range of divisional weight classes that exist within MMA and individual preference. Other researchers have reported similar findings of RWL in relation to time. Brito et al. [22] reported Brazilian combat athletes decrease 1.5 kg to 3.6 kg (3.3 lbs. to 7.9 lbs) in body weight during the week of competition. Whereas in this study, 8 athletes reported losing 5 kg to 6.8 kg (11 lbs. to 15 lbs) during the week leading to competition, well beyond the amount previously mentioned. Furthermore, 2 athletes reported losing 7.3 kg to 9.1 kg (16 lbs. to 20 lbs), and another 2 athletes reported to losing an astounding 11.8 kg (26 lbs) or more in the last week leading up to competition. In comparison, Coswig et al. [7] observed RWL from 1.1 kg to 7.4 kg (2.4lbs to 16.9lbs) a week prior to competition in 5 MMA athletes, and Matthews and Nicholas [24] also observed RWL from 1.4 kg to 5.6 kg (3lbs. to 12.3lbs.) a week prior to competition in MMA athletes from the United Kingdom.

MMA athletes are more likely to decrease weight in the last few days through hypohydration-inducing methods, including restricted fluid intake, training with sweat-suits, use of the sauna, and spitting [3]. In the present current study, the most frequently used methods of RWL included food restriction (82.6%), water loading (72.8%), sauna use (69.6%), increased training (69.6%), sweat-suit use (59.6%), and immersing in salt baths (29.3%). Similarly, previous studies have also reported these common methods of RWL [24, 26, 28]. These findings indicate that current professional MMA athletes are increasing the amount of weight lost in the last few days leading up to competition, which may place them at higher risks of hypohydration.

Dehydration in MMA athletes has been previously reported by Jetton et al. [4], using urine specific gravity which revealed 39% of the athletes were significantly dehydrated less than 24 h before competition. In addition, when investigating the effects of RWL in 17 amateur boxers, body weight loss was reduced by 1.7 to 5.6% 1 week before competition [25]. A trend following previous studies indicates the increased strategy of water loading. Water loading is the process in which athletes will decrease body water mass by increased urine production [26]. Athletes will drastically increase intake of water leading up to the finals days prior to competition and then restrict fluid and sodium intake to manipulate increased urine production [5, 26]. This fairly new method was reported amongst 67% of MMA athletes by Crighton et al. [5], 57% of MMA athletes by Matthews and Nicholas [24], and 72.8% of MMA athletes in our current study.

Other methods of RWL have remained consistent and may also indicate that MMA athletes are likely to use more than one method of RWL. Specifically, the common use of sweat-suits and saunas for RWL has remained to athletes in combat sports. When investigating 2638 international junior competitive wrestlers in 1994, 55% reported using sweat-suits, and 48.9% reported using saunas for RWL [18]. In addition, 50% of Brazilian combat athletes reported using both sweat-suits and saunas, and 43% of MMA athletes from the United Kingdom reported using both sweat-suits and saunas as well [24, 26–28]. In a more recent study, 51% of all combat athletes including MMA, boxing, Brazilian jiu jitsu, judo, tae kwon do, and muay thai reported using the sauna; however when looking at specifically MMA athletes, the percentage was reported higher at 76% [27]. This was similar to the use of sweat-suits for RWL within the same study, which was utilized by 43% of all combat athletes but 63% of MMA athletes specifically [27]. In our current study, 59% of MMA athletes reported the use of sweat-suits and 69% reported the use of saunas. The use of sweat-suits and saunas for RWL may provide evidence for dehydration as a main outcome of weight-cutting, as both methods focus on rapid depletion of body water [3, 6, 28].

This study also supports previous research indicating food restriction to be the most common method of RWL [23, 27, 28] In addition, our current study revealed that MMA athletes used the method of food restriction to reduce the most weight for their weight cuts compared to other methods of RWL. The current study found 19 MMA athletes indicated using the advice of a registered dietitian nutritionist for guidance on their weight cutting practices. Despite registered dietitian nutritionists being the most qualified professional to advise an athlete in terms of managing their body weight composition and having the adequate fuel for physical performances, they were amongst the lowest sources from which athletes sought advice.

To the authors knowledge, the present study is the first to report that professional MMA athletes who reported using the guidance of a registered dietitian nutritionist also used the fewest number of methods to cut weight. In addition, MMA athletes who used teammates as a resource but not a registered dietitian nutritionist also reported using more methods than those who used both or just a registered dietitian nutritionist. In this study, we were unable to make a direct comparison between those who only used a registered dietitian nutritionist and those who only used teammates because the number of athletes between groups without overlap were too unbalanced to conduct an ANOVA. This is novel because it is currently unclear if the number of methods used to cut weight has an impact on an MMA athletes’ ability to successfully make weight in a safe manner. Thus, it is also unclear to what effect using a registered dietitian nutritionist may have on a professional MMA athlete’s success to make weight for competition.

Although it is currently inconclusive if using the guidance of a registered dietitian nutritionist will lead to a more successful weight cut, the authors suggest head trainers, coaches, and MMA athletes consider using the guidance of a registered dietitian nutritionist for their expertise in regulating food and fluids for optimal physical performances. Employing the help of a registered dietitian nutritionist may reduce the likelihood of a professional MMA athlete to use inappropriate or unsafe measures for RWL. Future research should investigate the performance outcomes of professional MMA athletes who employ registered dietitian nutritionists for guidance with weight management for competition. Future research should also investigate if through the guidance of a registered dietitian nutritionist, MMA athletes may also be more likely to manage their weight continually, on a long-term basis, rather than only in preparation for an upcoming competition.

This study is not without limitations, the first being the use of self-reported measures, as athletes were not directly supervised to provide accurate and authentic information about their weight-cutting practices. The second is that the status of being a “professional” MMA athlete was not clearly defined, and thus athletes deemed “professional” is most likely varied in status and experience. The types of organizations that the athletes were competing in currently was not a measure obtained. Therefore, MMA athletes in top-tier organizations are more likely to afford professional services to help cut weight than those in less distinguished organizations. Lastly, professional female MMA athletes were not included in this study; inclusion of female athletes in future studies may depict a different scope to weight-cutting practices for female professional MMA athletes (Additional file 1).

## Conclusions

The findings of this study suggest that professional MMA athletes report undergoing RWL through various methods to make weight for competition. Professional MMA athletes report starting to reduce body weight at week 4 or 6 with the most amount of weight lost during the final week of training. Professional MMA athletes reported using various methods of RWL, where the highest reported were food restriction and water-loading. Most athletes reported undergoing RWL for competition without the guidance of a registered dietitian nutritionist. It is unclear what the effect of using a registered dietitian nutritionist may have on an MMA athletes’ ability to reduce weight is a safe and effective manner. Future research should seek to investigate if employing a registered dietitian nutritionist may lead to a higher rate of success for MMA athletes to make weight and/or improve performance. Furthermore, our study suggests that head coaches and trainers advise their MMA athletes to seek the guidance of a registered dietitian nutritionist for an upcoming weight-cut as they are still the most qualified professional to give accurate information on best practices for regulating food and fluids for physical performances.

## Additional file

**Additional file 1.** Appendix A: MMA questionnaire on habits of weight-cutting and sources of advice.

## Data Availability

The datasets used and analyzed during the current study are available from the corresponding author upon reasonable request.

## Abbreviations

MMA: Mixed martial arts
RWL: Rapid weight loss
