# Supplementary material for: Self-reported methods of weight cutting in professional mixed-martial artists: how much are they losing and who is advising them?
Source: J Int Soc Sports Nutr. 2019 Nov 12;16:52. doi: 10.1186/s12970-019-0320-9 (PMC6849211; doi:10.1186/s12970-019-0320-9)
Supplement: Supplementary file 1 — Additional file 1. Appendix A: MMA questionnaire on habits of weight-cutting and sources of advice. [file 12970_2019_320_MOESM1_ESM.docx]

**APPENDIX A.**

Q1. What country and state do you train most often?

Country _______________State________________

Q2. What is your gender? (Mark one answer)

- Male
- Female

Q3. What is your typical body weight in pounds (that you walk around in the off season)?

________________________________________________

Q4. What is your body weight today in pounds?

________________________________________________

Q5. If you were selected to obtain your body fat percentage, what was your percent body fat when the researchers measured it? If not please leave this section blank.

________________________________________________

Q6. How old are you? (Mark one answer)

- 18-23
- 24-28
- 29-34
- 35-40

Q7. How many years have you been competing as a professional MMA athlete? (Mark one answer)

- 0-3 years
- 4-6 years
- 7-9 years
- 10-12 years
- 13-15 years
- 16-18 years
- 19-21 years
- 22+ years

Q8. How many professional wins do you have as an MMA athlete? (Mark one answer)

- 0-3
- 4-6
- 7-9
- 10-12
- 13-15
- 16-18
- 19-21
- 22+

Q9. How many professional losses do you have as an MMA athlete? (Mark one answer)

- 0-3
- 4-6
- 7-9
- 10-12
- 13-15
- 16-18
- 19-21
- 22+

Q10. How many no contest/draws do you have as a professional MMA athlete? (Mark one answer)

- 0-3
- 4-6

Q11. How many professional MMA fights do you have in one year on average? (Mark one answer)

- 1-3
- 3-4
- 5-6
- 6+

Q12. What weight class do you fight in? (Mark one answer)

- atomweight
- flyweight
- bantamweight
- featherweight
- lightweight
- welterweight
- middleweight
- light heavyweight
- heavyweight

Q13. How often do you train during the off season (when you have more than 3 months until your next scheduled fight)? (Mark one answer)

- 0-2 days per week
- 3-4 days per week
- 4-5 days per week
- 6-7 days per week

Q14. On training days, how many hours per day do you train in the off season on average? (Mark one answer)

- 1
- 2
- 3
- 4
- 5+

Q15. How long is your training camp (formal training with coaches) when you are preparing for a fight?

- less than 6 weeks
- 6-8 weeks
- 8-10 weeks
- more than 10 weeks. If more, please indicate below how long in weeks.

________________________________________________

Q16. How often do you train during your season/training camp (when you have a fight scheduled within the next 3 months)? (Mark one answer)

- 1-2 days per week
- 3-4 days per week
- 4-5 days per week
- 6-7 days per week

Q17. On training days, how many hours per day do you train during the season/ training camp? (Mark one answer)

- 1
- 2
- 3
- 4
- 5+

Q18. Do you cut weight for your fights? (Mark one answer)

- Yes
- No, I don't want to
- No, I've never tried

Q19. On average how much weight do you cut? (Mark one answer)

- 0-2 pounds
- 3-5 pounds
- 6-10 pounds
- 11-15 pounds
- 16-20 pounds
- 21-25 pounds
- 26+ pounds

Q20. When do you start to cut weight for your fight? (Mark one answer)

- 1 week prior to the weigh in
- 2 weeks prior to the weigh in
- 3 weeks prior to the weigh in
- 4 weeks prior to the weigh in
- 5 weeks prior to the weigh in
- 6 weeks prior to the weigh in
- 7 weeks prior to the weigh in
- 8+ weeks prior to the weigh in
- I don't cut weight

Q21. When would you consider you drop the most weight before your weigh in? (Mark one answer)

- 2 weeks prior
- 1 week prior
- 5 days prior
- 3 days prior
- 48 hours prior
- 24 hours prior

Q22. Which methods do you use to cut weight? (Mark all that apply)

- food restriction
- sauna
- diuretics
- plastic sweat suit
- increased training
- water- diet (water loading followed by urination)
- vomiting or laxatives
- salt bath
- Other ________________________________________________

Q23. Following weigh-ins, how much weight do you put back on by the start of the fight? (Mark one answer)

- 0-2 pounds
- 3-5 pounds
- 6-10 pounds
- 11-15 pounds
- 16-20 pounds
- 21-25 pounds
- 26+ pounds

Q24. Do you have a professional nutritionist/dietitian that advises you for your weight cut? (Mark one answer)

- Yes, for all of my fights
- Some of my fights
- No

Q25. Do you have a professional nutritionist/dietitian that advises you during your off season? (Mark one answer)

- Yes, always
- Sometimes
- No

Q26. Where do you receive your nutrition advice? (Mark all that apply)

- Registered dietitian nutritionist
- Teammates
- Magazines
- Doctor
- Social Media
- Professional Organizations. Please describe ___________________
- Other _________________________________________________

Q27. Which supplements do you take? Any please indicate brand, approximate days/week, and dosage. (Mark all that apply)

- Creatine _________________________________________________
- Beta Alanine ______________________________________________
- Fish Oil __________________________________________________
- (HMB) Beta-Hydroxy-Beta-Methylbuterate _____________________
- Protein Powder ____________________________________________
- B-vitamins _______________________________________________
- Vitamin D ________________________________________________
- Coconut Oil (MCT oil) ______________________________________
- Thermogenics _____________________________________________
- Pre-workout supplements ____________________________________
- (BCAA) Branch chain amino acids ____________________________
- Multi-vitamins ____________________________________________
- Iron _____________________________________________________
- Other ____________________________________________________

Q28. How do you re-hydrate after you weight cut? (Mark all that apply)

- none. No specific re-hydration regimen
- Sports Drink/Pedialyte
- Coconut water
- electrolytes
- b-vitamins
- carbohydrate supplements
- protein powder
- Other. Please describe ______________________________

Q29. How many ounces do you typically rehydrate with immediately after your weight cut, within 1 hour of the weigh ins (1 cup =  8 oz)? (Mark one answer)

- 8-16 oz
- 17-24 oz
- 24-32 oz
- 33- 40 oz
- 40-64 oz
- 64+ oz

Q30. Approximately how many days does it take for you to go back to your starting body weight (before you started to cut weight) following your weight cut? (Mark one answer)

- 0-2 days
- 3-6 days
- 1 week
- 2 weeks
- 3 weeks
- 4 weeks
- 5 + weeks

Q31. Would you be interested in speaking to a nutritionist/dietitian that is a specialist in MMA nutrition programming over the computer or your smart device? (Mark one answer)

- Definitely yes
- Probably yes
- Might or might not
- Probably not
- Definitely not

Q32. What concerns do you have regarding your sport nutrition needs or weight cutting regimen as a professional MMA fighter?

- Answer Below: ________________________________________________
